# Abstracts and Gleanings

**Published:** 1886-07-20

**Authors:** 


### ABSTRACTS AND GLEANINGS.

**The Turks.**—The correspondent of the Iowa *State Medical Reporter* writing from Constantinople says: "I went the other day with Mr. Richardson and Mr. Sickles, two American friends, to Scutari, Asia Minor, to see the howling Dervishes who, in addition to doing many foolish and absurd things, attempt to cure the sick by the actions of a high saint whom they always have with them. The "meeting" is opened in a small old mosque. A fee is charged except from Mohamedans. There is a ring of about twenty-five feet in diameter. The high priest stands on one side and the Dervishes arrange themselves opposite to him in a line, each one having the skin of some animal upon which, for a considerable part of the service he kneels. They sing a horn'd howling sort of music (?) during the whole service, which is about three hours long. After remaining on their knees for about an hour with their heads upon the floor, they rise, walk around the room a few times and then commence the real scene. It is difficult to describe the extreme bodily motions. Such contortions and gyrations can hardly be imagined. They really seemed wild—sweat in great quantities would run from them and the exhaustion seemed impossible to bear, yet they kept it up for at least two hours. Towards the last of the service the healing of the sick commenced. It was a sort of clinic although not a word was spoken except the continued howling of the members. The patients were admitted or brought in on beds and placed before the high priest on the floor with abdomen down or up as he directed. The patients were of both sexes and of all ages, say from two years up to seventy. The high priest would walk over and then *stand* on the patient, small or large. I noticed that he stood so that the weight would be distributed over the pelvic arch. The right foot on the pubes or sacrum, according to which position the patient assumed, and the left are resting upon the abdomen or back. He would stand about a minute during which time he would go through some motion. Patients would get up crying, and hurry away. Sometimes he would stand on the joints in case of rheumatism or synovitis. In one instance he arranged several iu a row, belly down, and took several walks across them when the patients would get up and limp away. In some instances he would blow over them and pass his hands mysteriously in different directions. Water was brought and "loaded" with wonderful power for the sick who could not come.

Now what chance is there for medical science in a country where nearly all of the natural citizens believe in such nonsense which, by the way, is sustained by the government. It seems to me from what I have seen that the Koran has more real influence over the Mohamedan than the bible has over the Christian. The Turk takes pleasure in carrying out the instructions of Mohamed and it does not matter whether the duty is performed in public or in private it is done thoroughly. I have been led to make some inquiries into the multiple wife feature of the Turkish life and find that the harem is a Turk's home and he may have one wife or many. The poor man is restricted to a limited number, from one to four, but he may have concubines, women to whom he is not married. The Turk of rank or wealth may have as many wives as he chooses, and concubines or slaves besides. The present status of the harem is not owing to the great expense incurred in managing them, it is supported by only the rich and titled class—I mean the harem where more than one wife is kept. Each woman of the harem has many wants and slaves are usually owned as servants. The eunuch is the trusted party of the harem and is, as a rule, purchased from the Soudan country. There are two kinds of eunuch, each having a different value according to their qualities. They are prepared for eunuchs in infancy, one preparation is to remove the testicles. This is not the most satisfactory for the proprietor, because erections without the testicles occur and the eunuch has been found attempting to gratify the wants of the mistress. So a eunuch without either penis or testicles is the most valuable. The operation for removing both organs consists, in the infancy, of cutting all away with one sweep of the knife and then standing or burying the child in the sand, leaving the head above the surface. If he dies, all right, but if at the end of two days he survives he is taken out and all hemorrhage having ceased he is permitted to get well. It is stated, curious enough, that a contraction of the obscure mouth of the urethra does not occur. Those who have amputated the penis know how troublesome the meatus becomes when the membrane is not properly secured. Eunuch number one brings from one to three hundred dollars. Eunuch number two brings five hundred dollars. In color they are intensely black, usually tall, flat face, scarcely any nose except a couple of fluted pieces of flesh over a large everted upper lip, the lower lip rolling over towards the chin. The voice is soft and high pitched. These fellows have the freedom of the harem, and supply all of the wants possible to the wives or concubines. One would suppose that the offspring at the harem would be large with such facilities at command, but such is not the case. The main reason being that systematic prevention of conception, and when pregnancy has occurred, abortions are produced, of course not in every case, but in such cases as the master of harem directs. Turkish women generally incline largely to these practices, consequently the citizens of the empire are not increasing as they would under other conditions and circumstances. For preventing conception they use a piece of "bitter wood" an inch in diameter and about eight inches long. After the completion of the sexual act they proceed, with this cylinder of wood introduced into the cavity of the vagina, to mutilate the parts until free hemorrhage occurs when they consider themselves safe.

Should one of the wives of the harem have "twin boys" she immediately "passes to the head" and becomes the most important woman in the harem, even if the number should be large. The rule is that a woman is of no account unless she demonstrates her ability to bear children, and in the event of sterility her husband may send her away at any time. No knowing how much vaginal or uterine pathology these women have as a result of their cruel treatment of themselves, for examinations of the organs are very rarely permitted by the husband or owner of the concubine. When a man buys a wife he pays two-thirds down and he owes her or her father the remaining one-third so long as he may keep her, but should he send her away, which he can easily do, he must pay the other third. Should she go of her own accord, as she may do for cause, he need not pay the other third. When the woman leaves by discharge or otherwise she cannot marry for three months so that she may show that she is not pregnant. A man may marry a discharged wife twice without interference, but he cannot marry her a third time without her, in the meantime, having been married again. The custom of a third time marrying the same wife is interesting. For very slight offenses the wives are sent off and the man repents and wants her back. When the third time comes he is humiliated by having her marry some bought up or friendly selected party who rooms with her in a room under which the aspirant for the "third time" must spend the night alone. Are the conditions of purgatory less severe? The Turkish women are, as a rule, true to the Turk. There is not a house of prostitution conducted by Mohamedans in Constantinople. The English and other European representatives supply these immoral accommodations. There is much more that could be said concerning the Mohamedan Turk and his life which would be interesting, but my letter is already too long, so I will wait for another occasion.

**Asthma.**—Dr. Yandell (in *Practitoner and News*) writes: One night in July, 1865, I saw a well developed girl, six years old, in a sharp asthmatic seizure, which was soon relieved by a few doses of tincture of lobelia. I found that for two years before she had been subject to such attacks whenever she caught cold, and that the paroxysms had gradually grown more frequent, less and less "cold" being required to excite them. She was usually much worsted by a seizure, two or three days elapsing before she felt fully well again.

At my next visit I directed 10 grains of bromide potash to be given in a glass of seltzer water every morning on rising and at bed-time. To the latter dose was added the one hundred and twenty-fifth of a grain of sulphate of atropia. The mother was instructed as to the pathogenic effects of the medicines. Two days after it was found necessary to increase the bromide by 5 grains at a dose, which soon produced anæsthesia of the fauces, when the quantity was reduced to twelve grains, an amount which was not exceeded. Dryness of the throat and slight dilatation of the pupils followed after four days' use of the atropia. This medication was continued steadily for three months. Throughout the greater part of this time, the patient had iron and strychnia after food. She was required to live in the open air and take a cold sponge-bath daily. She was provided with a cough mixture containing a considerable quantity of opium, and her mother directed to use it on the appearance of the first symptoms of a cold. She had in the period named but two attacks of asthma, both slight. In the ninety days preceding the treatment, she had five attacks. The treatment was now suspended for a fortnight, when the weather growing cold—this was in November—it was resumed and continued for the succeeding four months. In that time she caught several slight colds, but had no asthma until March, when after a wetting in a sleet, she had a mild seizure that yielded to 5 grains of Dover's powder. This was her last attack. For the next four months the medicines were given fifteen days in each month, and then omitted until the following December, when they were given uninterruptedly for sixty days. Ten months having passed without a seizure, notwithstanding the patient had suffered several sharp catarrhal attacks in the time, further treatment was deemed unnecessary. It is proper to add that the patient made no change of house, and had practically the same surroundings during the entire time. She remains free from asthma to this day.

Since this case I have treated by the same method eight other cases of asthma in persons aged respectively three, ten years; two, eleven; one, twelve; one, thirteen, and one fourteen years old. All recovered but two, and in neither of these was the treatment fairly carried out by the parents. None were dismissed under fifteen months, while two were under treatment for two years.

In five of the nine cases, the disease was hereditary. Eight of the nine were unmistakably neurotic. Perhaps this fact may serve as an explanation of the success of the treatment.

**Experiments on a Decapitated Head.**—The French physicians, Drs. P. Reynard and P. Loge, had the opportunity to be present at a decapitation by the guillotine and to make experiments on the body from the moment of execution. They first observed a contraction of all the muscles of the face and of the body, suddenly beginning with the drop of the blade, and continuing for several minutes. During this time it was utterly impossible to flex the legs either in the hip or in the knee-joints, or to open the eyelids and to retain them open. In this respect, Prof. Martin, who reports the observations of Drs. R. and L. in the *Centrbl. f. d. Med. Wiss.*, 1885, November 14, remarks, that the exact place where the head was separated from the body should have been noted, for he once was present with Prof. Rossbach at a decapitation where the division occurred between the fourth and fifth cervical vertebra. The trunk dropped down lifeless and with every muscle relaxed, simply obeying the law of gravity, while the head continued for one and a half minutes to make dyspnœa-like respiratory motions, just like a person who when suffering from the most intense dyspnœa, would endeavor to breathe.

The first experiment, instituted by Drs. R. and L., thirty-two minutes after the drop of the fatal blade, consisted in electrical irritation of the vagus—never controlled by the observation of a differential hydromanameter which had been fastened in the trachea; at each irritation the index showed plainly a movement proving a diminution in the volume of the lung; both pleural cavities were well expanded. Direct irritation of the lung-tissue had the same effect. When thirty-five minutes after the execution the abdominal cavity was opened, the intestines seemed in a state of rest; contact with the air made no impression on them. Both vagi were now irritated, and the whole intestinal canal, from the stomach (which was empty) to the transverse colon, at once began active motion. The stomach was next opened; at every irritation of the vagi the mucous membrane folded itself, and was covered all over its whole surface with minute drops of gastric juice.

Though all reflex experiments were started immediately after the removal of the body from the guillotine, no reflex movements could be produced by the most powerful electrical irritation of the spinal medulla, after the opening of the canal, was also fruitless. The only reflex observed was a slight contraction of the pupils after their sudden exposure to strong light.—*Medical and Surgical Reporter.*

**New Method of Treating Whooping-Cough.**—Dr. Barlow gives the following conclusions:

"1. That whooping-cough is to be classed among the diseases which are caused by the irritation excited by the presence of parasites. 2. That it is due to the presence of micrococci, which proliferate in large numbers upon the lining membrane of the larynx and pharynx, and which infiltrate the epithelial cells, which seem to be the preferential seat of their growth and increase. 3. That resorcine, in a solution of the strength of 1 or 2 per cent., applied directly to the mucous surfaces concerned, has been found, in all cases in which it has been employed and the results watched, to rapidly reduce the number of chinks, to reduce their intensity, and finally to lead to the cure of the disease." This last word "cure" is a bold one, but if the first and second proportions are held to be proven, then there can be little doubt that it is properly used; for if there are no other diseases *cured*, yet certainly those which are due to the irritation of a parasite are cured when that parasite is destroyed.

"Lastly, the mode of application by means of a brush or swab is not the only mode in which it may be made; perhaps by some of the recently used instruments for applying medicated spray to the interior of the larynx and the adjacent parts of the throat (as that recently described by Dr. Miller, of Windsor, and by Dr. Hodgkinson) it may be applied yet more effectually than by the methods adopted by Professor Moncorvo and myself. And, again, it is not finally proven that resorcine is the best application that may be found. Perhaps in its near congener, orcine, we may have a still better agent for this purpose, for we find in a note by J. J. Andeer, in the *Gazetta Medica Italiana-Lombardia* of July 4, 1885, that "Orcine possesses almost all the physical properties of resorcine; but while the latter is caustic in large doses, orcine is never more than astringent and antiseptic."

The treatment, as will be seen, though announced as a new one, is in reality only carrying out more systematically the suggestions of previous physicians. Thus, as Dr. Barlow says: "Tordeus has used the benzoate of soda; Keuster, inhalations of thymol; Huebner, salicylate of soda; Hildebrandt, inhalations of petroleum vapor, etc. Resorcine was selected by Moncorvo because of its innocuousness."

The specific treatment of whooping-cough has always run in two lines, viz, toward antispasmodics, like the bromides and belladonna, and toward germicides, like carbolic acid and resorcine. That we shall be able, as a rule, to apply germicides so thoroughly as to be specifically effective remains to be seen. It is certain that in antispasmodics and sedatives the best results have so far been obtained, and belladonna and the bromides still hold their place.—*N. Y. Medical Record.*

**Gonorrhœa.**—The editor of the *Medical World* used in an obstinate case the following ointment, applied in small quantities to the glans penis, beneath the prepuce, on each side of the frenum:

| | | |
|---|---|---|
| ℞. | Morphine | 5 grains, |
| | Lanolin (wood fat) | $\frac{1}{2}$ drachm. |

He then combined muriate of cocaine with some of the above mixture, so that about one-fourth of a grain of cocaine would be used at each application. In a few hours the penis was found to be perfectly numb, discharge ceased, and all symptoms relieved. Continuation of this treatment soon effected a cure. The modus operandi is as follows: The diseased tissues being anæsthetized, they are robbed of pain and sensibility, thus being free from impressions or disturbance of any kind; cocaine causes vessels to contract, thus removing congestion and inflammation.

Thus it seems that we have an efficient and easily managed mode of treatment for most of the local diseases of the genito-urinary apparatus; if begun promptly we believe thet it will abort gonorrhœa; it will certainly cure urethral and vesical irritability immediately, and the cure may be permanent when not dependent upon stone or other mechanical causes of irritation.

Without waiting to make other tests, we hasten to give the idea to the profession, so that many thousands may make tests and report results upon what seems to be a very promising mode of treatment. Of course, the part should be cleansed before each application. Be sure to have the mixture strong enough to produce decided effect. Try, and report in full as to strength of mixture used, amount, and frequency, condition before use, and results. Details are necessary to the establishment of a new mode of treatment, and this is now as far as we know.

**A Classical Remedy for Hiccough.**—Dr. A. G. Gibson calls attention to the old Hippocratic aphorism, "Sneezing occurring after hiccough removes the hiccough," and suggests, in cases of hiccough, the production of sneezing by tickling the nostrils, and he tells us that he has in this way been very successful in the arresting of this disagreeable affection. Hiccough, as well as sneezing, is one of the specially modified respiratory movements, and it is quite in accordance with what we know of the transference of nervous action that the spasmodic contractions of the diaphragm should cease on the induction of the explosive expirations which constitute the acts of sneezing. There is one point, however, which deserves special mention : It is not necessary that the stimulus applied to the nose be followed by sneezing. The application of a gentle irritant to the nasal mucous membrane may be quite enough to put a stop to the hiccough, by diverting the nervous energy into other channels, although it mav not be of sufficient power to induce sneezing.—*Lancet and Clinic.*

**Chinese Medical Missions.**—Unlike Japan, which has rapidly absorbed European medical and biological science, and is training men of first-rate knowledge and considerable original research, China is still in outer darkness. Chinese medicine is a byword; Chinese surgery is almost non-existent; it is not surprising, therefore, to find that the Celestials are willing to take advantage of European skill, and attend the mission hospitals at Swatow and Ung-Kang-Phu in considerable numbers. The hospital arrangements for in-patients appear to be delightfully simple. "Scarcely a day passes," writes Dr. P. B. Cousland, in his annual report, "but little groups of, perhaps, husband and wife, mother and children, or a company of fellow villagers, may be seen coming into the hospital courtyard, with their baskets of the all necessary rice, a few cooking utensils and bed-mats, and, perhaps, a mosquito curtain, depositing them in a convenient corner until the janitor, or matron, has assigned to them their proper ward." Though there were 3,867 in-patients during the year, the only expenditure of the hospital in food seems to have been a sum of ninety dollars for "rice and cash tickets," so that the expenses are limited to the cost of drugs, and the wages of assistants and servants. The assistants, who do duty as house-surgeons and dispensers, are natives. The objection to submitting to amputation is very strong, and Dr. McPhun, in his report from Ung-Kang-Phu, where 2,620 patients were treated, complains that the majority of his patients were either incurably ill, or suffering from external diseases, because the Chinaman fears that internal remedies will work magically upon the heart to turn it from his ancestral religion. A very large proportion of the patients treated at the mission hospitals are suffering from diseases of the eye, the most common being trichiasis, and granular ophthalmia with its consequences. The number of opium-smokers treated at Swatow alone was 649, but the prospect of permanent cure is not spoken of hopefully ; many run away, unable to endure the misery of the first few days, and but few remain long enough to undergo a course of tonic treatment, which is considered to be essential.—*Medical and Surgical Reporter.*

**Disguising the Taste of Quinine.**—Query: What is the best method for disguising the taste of quinine without interfering with its therapeutic action?

After enumerating the various methods employed for this purpose the writer says:

"A superior method is one we would recommend. It consists of trituating equal parts of sulphate of quinine and sugar of milk with twice the weight of crystallized phosphate of sodium, and this method can be further improved upon by the addition of saccharin in proportion of $\frac{1}{4}$ or $\frac{1}{3}$ grain to each grain quinine. The quinine when taken in this manner is, by double decomposition, changed to a phosphate, which is soluble in only 750 parts of water. When taken it is slightly bitter to the taste, and the phosphate of quinine in, so to say, *statu nascenti*, will be quickly dissolved in the gastric juice. Gelatin capsules are often objected to by children, and sometimes become insoluble. We have known them, as well as compressed quinine pills, to pass undissolved through the intestines. We know of no more elegant nor practical form of dispensing the sulphate of quinine in powder form than the inclosing in wafers. The wafers, when properly stored away and protected from insects, will keep in all climates; if carefully filled and sealed they prove superior to any modern product of *la pharmacie elegante*. After long experiments we have, however, found the above described method of employing phosphate of sodium and the lately discovered saccharin, to be useful, and the simplest." —*National Druggist.*

**Prescriptions for Sciatica.**—Dr. Eldordin recommends cupping sacrum, give 1 drop aloin tig. for three days, quinine 30 grs., iod. potass. $\frac{1}{2}$ drachm, vin. colchici 1 ounce, aquæ 6 ounces. Sig.: One teaspoonful three times per day, then give mixture of tinct. nux vom. 10 drops three times a day.

Dr. McClintock recommends acapuncture, inject, one-half drachm cold water. Repeat daily—into sciatic nerve, if possible.

Dr. Ebert recommends calomel, iodine over seat of pain, Dover's powder to produce quiet, quinine in 2-grain doses every two hours.

Dr. Sullivan recommends cold water 4 ounces, 2 grains permanganate potass. hypodermically. Repeat two or three times a day.

Dr. R. L. Smith recommends 10 drops ether, $\frac{1}{4}$ grain morphine hypodermically over seat of pain.

Dr. Hillman recommends paste of quinine and castor oil, friction over nerve; lemon juice, if tongue is red and pointed.

Dr. Anderson recommends 20-drop doses balsam copaiba every two or three hours.

Dr. Cone recommends minute doses tartar emetic, and applying tobacco leaves over seat of pain.

Dr. Goslin recommends hypodermic injections into the tissues, 1-30 grain sulph. atropia once in twenty-four hours.

I fear my article will be too lengthy, and will stop. If the profession desires the balance of the prescriptions, I will give them, if requested.—*Dr. Webb in Medical Brief.*

**Earache.**—A short time ago we had a case of intense earache under our charge. The patient was a married lady aged thirty-one. The entrance to the external meatus seemed greatly swollen, and was very tender to the touch. The patient apparently suffered great agony. From 4 to 6 drops of a two per cent. solution of muriate of cocaine were dropped on a piece of absorbent cotton, and the latter pushed into the meatus. By the time we had suceeded in placing the cotton as we wished, all pain had ceased. The patient was advised to repeat the procedure herself every three to four hours. Two days later the symptoms had all disappeared, without any visible discharge.

A little boy aged three was suffering from earache. For two hours the little patient rolled around in bed, the severe pain preventing him from sleeping. When we arrived, the boy did not permit us to touch his ear; and we had considerable trouble in persuading him to allow us to apply the cotton, wet with a two per cent. cocaine solution, to his ear. The pain also yielded in this case at once, and the earache did not return.—*Philadelphia Reporter.*

**Rapid Cure of Suppuration of Middle Ear of Thirty-five Years' Standing.**—The *Medicel Press* tells us that Dr. G. B. F. Brunetti, of Venice, reports the following:

The patient, a physician, æt. forty, had suffered from offensive otorrhœa since his fifth year. Hearing power had gradually sunk to a minimum. The watch could only be heard on contact. The tympanum was absent on both sides. The ossicles were present on both sides; on the left they were incompletely ankylosed. After cleansing the auditory passage and middle ear, iodoform and sp. vin. rect. were employed, and in two days the stench disappeared; after eight days a few drops only of puss escaped. For eleven days a 0.5 per cent. solution zinci sulph. was used, and then the iodoform and alcohol again. In one month the patient was discharged, the vegetations in the tympanic cavity which were at first present had disappeared; its coverings had become healthy looking. Whispering could be heard at one metre distance, and the watch at 65-200 (left) and 30-200 (right) of normal.—*Quarterly Compendium.*

**White of an Egg in Obstinate Diarrhœa.**—From the *Allg. Med. Cent. Zeit.* we learn that Celli has recently called attention to the curative properties of the albumen of hen's eggs in severe diarrhœal affections. In a discussion before a medical society at Rome, he advocated its use, and related two cases of chronic enteritis and diarrhœa, which, having resisted all treatment, speedily made complete recoveries under the use of egg-albumen. The same diet is strongly recommended in the diarrhœa accompanying febrile cachexia and in that of phthisis. In two cases of diarrhœa dependent upon tertiary syphillis, it was found of no avail. On post-mortem examination, diffuse amyloid degeneration of the arterioles of the villi was found in these cases. The whites of eight or ten eggs are beaten up and made into an emulsion with a pint of water. This is to be taken in divided quantities during the day. More may be given if desired. The insipid taste can be improved with lemon, anise, or sugar. In case o' colic, a few drops of tincture of opium may be added.—*Medical and Surgical Reporter.*

**Treatment of Acute Rheumatism.**—The last number *Russkaya Meditsina* contains a communication from Dr L. Grinevitski, of Rostoff-on-the-Don, who writes that for more than twenty years he has treated acute articular rheumatism with nitrate of potash. 2 drachms being given daily in raspberry syrup, 10 grains every two hours. Together with this internal medication he prescribes an ointment for use morning and evening of the following composition:

℞. Olei hyosc.................................℥ 1,

Ung. hydrarg. cinerei...........................ʒ 2,

Ext. acon.......................................ʒ 1.

He has tried all ordinary remedies, and finds that on the whole this plan of treatment is more satisfactory than any other, being especially valuable in those cases where salicylates fail to give relief. Generally the disease is brought to an end in from one to two weeks, according to its severity and the time the treatment was commenced. When commenced at the onset of the attack, and before more than one joint was affected, the others were usually spared altogether.—*Lancet*, May 22, 1886.

**Sparteine at Home.**—Dr. Thomas H. Buckler, of Clifton, Md., writes to the Boston *Medical and Surgical Journal*: The expensive Sparteine lately recommended by German See for cardiac weakness, is prepared from Scotch broom—*spartium scoparium*—the *Planta Genista* or emblem of the Plantaganets, which grows in many parts of the thirteen original States, on sterile soil. It was brought over here by the Scotch and English to prevent the washing of gravelly roads and gutters. It should be cut and gathered at this season and dried like hay. Its active principle is extremely soluble in water, and two ounces of ground or contused stems to a quart of boiling water, a wine-glassful for a dose, every eight hours, is equivalent to a grain and a half of the prepared gum used by Dr. See. This remedy is useful not only in failure of the cardiac ganglia but as a tonic to the organic and vaso-motor nerves in whatever part of the body congestions occur from loss of power in them. I have used this agent in the form of infusion for half a century and with marked advantage in many cases.—*Maryland Medical Journal*

**Cotton-Root Bark in Epilepsy.**—Cotton-root bark has been tried by Dr. Clevenger in some of the nervous derangements in which ergot has been heretofore mainly employed, and in his reports in the *Journal of Nervous and Mental Diseases*, he says that he has found it valuable in epilepsy and paretic dementia, when given in doses about three-fourths as large as the dose of ergot in the same diseases. He says it is less liable to produce gastric disturbance, and acts more rapidly than ergot, but in this we believe he is mistaken, as fluid extract of ergot is not nauseating if properly made to avoid decomposition and consequent formation of trimethylamine, which latter substance renders the preparation nauseous.—*National Druggist.*

**Some Forebodings of Incipient Insanity.**—

1. Irritability and tendency to take offense.
2. Moroseness and silence, or sometimes fault-finding with servants.
3. Suspicion and jealousy of best friends.
4. Impairment of memory, forgetting hours of meals.
5. Inattention to exercise and state of bowels.
6. Neglect of personal appearance.
7. Altered facial expression, notably in melancholia, with marked furrows.
8. Prominence and brilliancy of cornæ, in hysterical and puerperal mania.

*Bodily Symptoms.*—

1. Harsh, dry skin as a rule, though sometimes perspiring.
2. Sometimes a peculiar odor.
3. Coated tongue, with offensive breath.
4. Constipation and feeble circulation.
5. Headache and palor of face.
6. Sexual appetite, either in abeyance or abnormally strong.
7. Frequent suppression of menses in females.
8. Subjective deafness, or abnormal auditory sensations.
9. Altered conversational style, and talking to ones-self.
10. Delusions and illusions later on.—*Medical World.*

**Pilocarpin in Phneumonia.**—The Paris correspondent of the *British Medical Journal*, tells us Dr. Humbert Molliere has successfully treated double pneumonia with pilocarpin; the patient, exhausted by dysentery and albuminuria, was attacked with pneumonia in both lungs, and the intestinal disturbance was greatly aggravated. A centigramme of pilocarpin was injected; the respiratory movements fell from forty eight a minute to twenty-four, and dyspnœa was much relieved; four hours later, a fresh injection was made, and was repeated the next morning; each injection was followed by profuse sweats and salivation, and dyspnœa was greatly relieved. The patient rapidly recovered. In administering pilocarpin, M. Molliere was guided by former experience. An elderly man with uremia, accompanied with dyspnœa and delirium, who seemed dying, was greatly relieved, and ultimately cured by a similar treatment. Dr. Molliere describes another case. A young woman with a comatose form of uremia and renal lesion, following a cardiac affection, was freed from the comatose condition by injections of pilocarpin.—*Medical and Surgical Reporter.*

**Brucine.**—Dr. Mays, of Philapelphia, reports that he finds a 5 per cent. solution of pure brucine an efficient local anæsthetic. He has applied it successfully in a case of toothache in a decayed tooth, and for relief of the burning produced in the mouth by Cayenne pepper. Externally, it diminishes sensation, and has been found to relieve itching—also the pain produced by a mustard poultice, and the itching by Croton oil application.

Dr. Zeiss tried a 3 per cent. solution for relief of pain from furncles in the external auditory canal. The pain was mitigated in two to four minutes, and did not return for several hours. In the pain of suppurative otitis, it gave some relief. He had found it especially useful in mitigating pain produced by applications to the throat and nasal passages of iodine, sulphate of copper, nitrate of silver, etc. In burns he had found it of no value.

Dr. Squibb highly lauds creosote water as an anæsthetic application to burns and scalds, and the remedy is one capable of more extended uses.—*American Lancet.*

**Membranous Croup.**—In the February *Brief*, page 71, a doctor wishes a more successful treatment of membranous croup than he has been using. I am sorry for him, that he has such poor success. I think, doctor, since your usual remedies all fail, you had better try some of the simple remedies. Let me give a few: One drachm of strong alum water has often stopped the disease in less than five minutes; pulverized alum and sugar, equal parts; dose, a teaspoonful; pulverized alum mixed with white of egg; pulverized alum with molasses often repeated; lard two parts, molasses one part, or nearly in this proportion; give it freely every ten minutes until the breathing becomes easy. If the child vomits continue the remedy and also keep the throat externally well covered with lard, and lay on flannel to exclude the air as much as possible.

This last named remedy has been tested in hundreds of cases and never yet known to fail. Please try them; they will do no harm.—*Dr. P. C. Smith in Medical Brief.*

**Chorea.**—I wish to report a case of choera. A boy twelve years old was so affected with it, that he could neither walk nor feed himself. About one week after he was taken sick he became so nervous that they had to hold him on the bed. A physician was sent for; he put him on calomel and rhei to move his bowels; also put him on comp. tinct. avena 20 drops three times a day, which relieved him to some extent, but did not cure. We then put him on strychnine 3 grains to 2 ounces water. Gave him from 6 to 10 drops three times a day. Also pustulated the spine from cervical to sacrum with croton oil, which made a cure in a few days. The boy is all right and at work.—*Dr. S. J. Terry in Med. Brief.*

**Hot Flashes.**—A writer in *Medical Brief* says: I have noticed several inquiries, recently, as to the most satisfactory treatment of the hot flashes and congestions accompanying and following the change of life. I have, during the past year, treated several of these cases, and in every case the following has given speedy, and almost immediate, relief, viz.: Acidi sulphuric diluti 4 ounces. Sig.: Fifteen to twenty drops in a wine glass of water three times a day.

**The Incompatibility of Calomel and Bromide of Potassium.**—Vigier (*Gaz. Hebdom. de Med. et de Chir.*, May 6, 1886), remarks that calomel is decomposed on the addition of potassium bromide, although more slowly than when the iodide is added. Nobody, he thinks, would give the drugs within five or six hours of each other, but it might happen, for example, in a case of infantile convulsions, that two practitioners being called in quick succession, the second one might order one of these drugs after the other had been given by the advice of the first one. He gives the caution, therefore, that in such cases their incompatibility should be borne in mind.—*New York Medical Journal*, May 22, 1886.

**The Digestibility of Various Kinds of Foods According to Vanderbeck.**—*Meats.*—Easy to digest: Mutton, venison, hare, sweetbread, chicken, turkey, partridge, pheasant, grouse, beef. Hard to digest: Pork, veal, goose, liver, heart, brain, lamb, duck, salt meat, sausage.

*Fish.*—Easy: Turbot, haddock, flounder, sole, oysters, trout, pike. Hard: Mackerel, eels, salmon, herring, salt fish, lobster, crabs, mussels, cod.

*Vegetables.*—Easy: Asparagus, French beans, cauliflower, beets, potatoes, lettuce. Hard: Artichoke, celery, spinach, boiled cabbage.

*Fruits, etc.*—Easy: Baked apples, oranges, grapess, strawberries, peaches, cocoa, coffee, black tea, claret. Hard: Apples, currants, raspberries, apricots, pears, plums, cherries, pineapples, chocolate, pickles, beer.—*Journal of Commerce.*

**Cause of Fever.**—Says Dr. Elliott, in *N. O. Medical Journal:* Fever results from a depression of that nervous center which controls the distribution of energy within the body, on account of which depression tissue-building ceases, and the energy destined to perform that act passes off into the lower form of heat. From this same failure of nerve power, and from the heat resulting, tissue-destruction in the body is usually increased.

**Salicylate of Soda in Sick Headache.**—The dose of salicylate which Dr. Haig uses is two to three grains every quarter or half hour for three or four doses or more, as recommended by Dr. Brunton, and begun when the headache first comes on; this is sufficient. A patient might carry ʒ i of the powder in his pocket and take a little when a headache threatens, and he would soon learn to judge the proper doses by sight.—*Exchange.*

**A Chicago** doctor who sat so heavily muffled in a street car as to completely conceal his identity, was treated to a scrap of conversation at his expense, which quite amused him. "Do you know Dr.——?" asked one passenger of another. "All I know of him is that he snatched an aunt of mine from the grave last summer," was the reply. "Did he, indeed? Well he must be a good doctor. What ailed your aunt?" "Oh, she was dead and buried, you know."

**The** patients bitten by the rabid wolf, who were brought to Pasteur from Russia, seem to have been but slightly benefitted by the treatment, several of the number having already died. Pasteur holds that the rabietic virus as it exists in the wolf is of a different nature and more virulent than that found in the dog.—*Med. Age.*

**Antipyrin in Phthisis at Davos.**—Dr. Hoedmaker, a Dutch physician, practicing at Davos Platz, has made a number of observations on the effect of antipyrin on phthisical patients, from which he has come to the conclusion that patients not treated with antipyrin are more comfortable than those who are taking it, though these latter have less fever. He has found salicylic acid most valuable in phthisis, especially when combined with arsenic. —*Lancet.*

**Jenner** was much given to versification. On one occasion, says the *Cincinnati Lancet*, he sent a brace of ducks, with the following lines, to one of his patients :

"I've despatched, my dear madame, this scrap of a letter
To say that Miss Dogby is very much better.
A regular doctor no longer she lacks,
And therefore I've sent her a couple of quacks."

**Hystero-Catalepsy in a Male Patient.**—Dr. A. McLane recently, according to the New York *Medical Journal*, cured a case of persistent hystero-epilepsy in a man thirty-five years of age, by squeezing the patient's testicles firmly in his hand. The patient, he says, awakened as if from a dream, with evident pain in his head and the usual cry. From that moment he had no return of the catalepsy, and becoming perfectly natural, he convalesced rapidly.

**A Specialist** in throat troubles was called to treat a Boston lady who manifested so much interest in his surgical instruments that he explained their uses to her. "This laryngoscope," said he, "is fiitted with small mirrors and an electric light: the interior of your throat will be seen by me as clearly as the exterior; you would be surprised to know how far down we can see with an instrument of this kind." The operation over, the lady appeared somewhat agitated. "Poor girl," said her sister, who was present; "it must have been very painful." "Oh, no, not that, not that," whispered the Boston lady; "but just as he fixed his instrument in place I remembered that I had a hole in my stocking."—*Journal of Reconstructives.*

**Alterative Medication.**—"Goot morning, mein friend. How you feel dese morning?"

Patient—"O Doctor! splendid, such an appetite: could eat any thing."

Doctor—"So, so—Never mind, I zoon give you somedings to dook away all dot."—*Ibid.*

**The** *Indian Medical Gazette* advocates the use of condemned criminals for cholera experiments. The criminals who have been heard from say they don't want any such funny business.

